# Identification of Changes in Rumination Behavior Registered with an Online Sensor System in Cows with Subclinical Mastitis

**DOI:** 10.3390/vetsci9090454

**Published:** 2022-08-24

**Authors:** Ramūnas Antanaitis, Vida Juozaitienė, Dovilė Malašauskienė, Mindaugas Televičius, Mingaudas Urbutis, Arūnas Rutkaukas, Greta Šertvytytė, Walter Baumgartner

**Affiliations:** 1 Large Animal Clinic, Veterinary Academy, Lithuanian University of Health Sciences, Tilžės Str. 18, LT-47181 Kaunas, Lithuania; 2 Department of Biology, Faculty of Natural Sciences, Vytautas Magnus University, K. Donelaičio 58, LT-44248 Kaunas, Lithuania; 3 University Clinic for Ruminants, University of Veterinary Medicine, Veterinaerplatz 1, A-1210 Vienna, Austria

**Keywords:** subclinical mastitis, rumination, activity, early diagnosis, precision agriculture

## Abstract

**Simple Summary:**

The aim of this study is to determine if there is a relationship between subclinical mastitis and rumination behavior, as measured by an online sensor system. According to the findings of our research, rumination time and rumination chews can act as biomarkers of subclinical mastitis. Moreover, changes in rumination behavior could be used to identify subclinical mastitis in dairy cows. Recognizing affected cows and treating udder inflammation at an early stage could be extremely beneficial to farmers. For the identification of subclinical mastitis, veterinarians and farmers could consider using an online sensor system that registers rumination behavior biomarkers. This system would allow early identification of sick cows and reduce economic losses to the farm caused by this disease.

**Abstract:**

The aim of the present study is to determine the relationship between subclinical mastitis and rumination behavior registered with an online sensor system. Based on the findings of the general clinical examination of 650 milking cows, 10 cows with subclinical mastitis (SCM) and 10 clinically healthy cows (HG) were selected (without clinical signs of any diseases). Rumination behavior biomarkers were registered with RumiWatch noseband sensors (RWS; ITIN + HOCH GmbH, Fütterungstechnik, Liestal, Switzerland). Sensors were implanted on the first day after calving. Data from the RWS 13 days before diagnosis of SCM and 13 days after diagnosis of SCM were compared with HG data from the same period. Healthy cows were checked alongside SCM cows on the same days. In our study, we found that healthy cows spent more time engaging in rumination and drinking (*p* < 0.05) and also had more boluses per rumination. Moreover, among cows with subclinical mastitis, rumination time from day 4 to day 0 decreased by 60.91%, drinking time decreased by 48.47%, and the number of boluses per rumination decreased by 8.67% (*p* < 0.05). The results indicate that subclinical affects time and rumination chews registered with sensor systems. However, additional studies with larger numbers of animals are required to confirm these results. Furthermore, the impact of heat stress, estrus, and other effects on rumination behavior biomarkers should be evaluated.

## 1. Introduction 

Mastitis is defined as an inflammatory condition of the mammary gland caused primarily by bacteria [1]. Bovine mastitis, specifically subclinical mastitis (SCM), is one of the most common and expensive diseases in the global dairy business. Understanding the prevalence and spatial distribution of bovine SCM, as well as the risk factors related to this disease, could aid in disease prevention and control [2]. Clinical symptoms of mammary gland inflammation include changes in milk and udder features, systemic indicators of disease, and higher SCC in milk [3]. Cows with SCC levels greater than 200,000 cells/mL are considered to have subclinical mastitis. SCM, like in conventional dairies, poses a significant risk to organic dairy animals. It was found that the incidence of SCM in cows under organic management is comparable to or higher than that in cows belonging to conventional herds [4]. In cows suffering from negative energy and protein balance in early lactation, innate immune function and inflammatory regulation are impaired, which is critical to the development of mastitis [5]. When detected and treated during the first 21 days of lactation, the clinical disease can have long-term impacts on lactation performance, reproduction, and the culling of dairy cows, all of which contribute to negative repercussions for dairy herd sustainability, such as health problems [6].

Milk parameters (such as somatic cell count (SCC), l-lactate dehydrogenase, milk lactose, protein, fat, milk pH, and temperature haptoglobulin) and blood serum indicators (minerals (Cl, Ca, and Na), lactate, acute phase proteins, amyloid A, C-reactive protein, plasma-carotene, and ceruloplasmin) are examples of biomarkers used for subclinical mastitis detection [7]. Electrical conductivity (EC) is also increasingly being used to identify mastitis. However, the results of this method are contradictory. The accuracy necessary to detect infected quarters cannot be provided by EC alone. Incorporating additional information (e.g., milk yield, milk flow, and quantity of incomplete milkings) may improve detection accuracy and the ability to detect the early beginnings of mastitis [8]. Wang et al. [9] found that bta-miR-146a and bta-miR-146b expression levels were significantly higher in bovine mammary tissues infected with subclinical, clinical, and experimental mastitis. Multiple immune processes involve potential target genes. These findings point to both miRNAs having regulatory roles in bovine inflammatory responses in mammary tissue.

For the early detection of mastitis in automatic milking system (AMS) herds, various data and alternative methodologies must be combined [8]. Data from sensor systems can be used alone or in conjunction with established health-monitoring techniques to detect cows suffering from health problems [10,11]. Monitoring of behavioral and health markers may enable the detection of subtle changes prior to the appearance of obvious clinical indications. Data preceding diagnoses are generally more valuable for earlier diagnosis and intervention if they can forecast risk or detect disease more efficiently than observations using clinical indicators [12]. Further studies are needed, however, to develop criteria for the identification and prevention of diseases based on data from an automatic health monitoring system [13]. Most diseases after calving, such as metritis, ketosis, mastitis, and others, can alter an animal’s walking behavior [14]. Early detection is critical for decreasing the deleterious effects of mastitis [15]. Among the methods used to diagnose this condition, rumination sensors have proven to be efficient in detecting mastitis and other diseases in their early stages [15,16,17]. RWS has been tested in dairy herds around the world for confinement and grazing and have the potential to be used as a standard to validate other animal behavior technologies [18]. Benaissa et al. [19] investigated algorithms for feeding and rumination behavior and discovered that a neck-mounted accelerometer outperformed RWS in terms of accuracy. Pereira et al. [18] applied a RumiWatch noseband sensor to evaluate the Smartbow ear tag sensor (Smartbow GmbH, Weibern, Austria) among Minnesota and Irish grazing herds and found good correlations between grazing behavior and visual observations.

The RumiWatch was designed and tested effectively as a scientific monitoring device for automated measures of behavior of rumination and activities [20]. The high to extremely high correlations between direct observations and sensor data show that the RumiWatch noseband sensor was designed and validated successfully as a scientific monitoring device for the automated detection of rumination and eating activities in stable-fed dairy cows [20]. Previously, we evaluated the association between SCK, locomotion behavior (feeding time with head up, feeding time with head down, frequency of switching between activities, and walking activity), and ambient temperature (average, minimal, and maximal temperature) [21]. Cows with subclinical ketosis were shown to change rumination and activity behavior. Lower minimum and maximum ambient temperatures were, moreover, linked to the onset of SCK [22].

According to the literature, we hypothesized that subclinical mastitis would affect rumination behavior in cows (such as rumination time, eating time, drinking time, rumination chews, eating chews, drinking gulps, and boluses) registered with sensor systems. To explore this hypothesis, we set objectives to determine changes in rumination behavior (such as rumination time, eating time, drinking time, rumination chews, eating chews, drinking gulps, and boluses) registered with an online sensor system in cows with subclinical mastitis.

## 2. Materials and Methods

### 2.1. Ethical Approval

The study was conducted in accordance with the Animal Welfare and Protection Act of the Republic of Lithuania (No. 108-2728; 2012, No. 122-6126). PK016965 was the study’s approval number.

### 2.2. Location and Animals

This investigation was conducted between 1 July and 15 December 2021, at the Lithuanian University of Health Sciences and on one Lithuanian dairy farm (54.9754° N, 23.7684° E). The average milk production per cow was 10,500 kg per year.

Since mastitis is a disease that is influenced by many factors, when selecting cows for this study, we paid special attention to the homogeneity of the samples so that all factors and external processes (such as heat stress, estrus, and changes to feeding rations) with potential to affect the data remained constant throughout the study period. Cows with these factors were excluded from the study.

The current examination comprised 20 cows out of a herd of 650 (on average, 2.58 ± 0.34 days for lactation and 187.65 ± 2.6 days for milk).

The selected cows average milk yield (MY) was 37.6 ± 0.3 kg, with milk fat (MF) of 4.31 ± 0.04%, milk protein (MP) of 3.51 ± 0.02%, a milk fat-to-protein ratio (F/P) of 1.23 ± 0.01, milk lactose (ML) of 4.60 ± 0.01%, milk urea (MU) of 27.02 ± 0.15 mg/dL, and milk somatic cell count (SCC) of 286.65± 35 thousand cells/mL. The cows were kept in a free housing system and fed a total mixed ration (TMR) twice a day at a set time of year, balanced to fulfill the physiological needs of a 550 kg Holstein cow producing 38 kg milk per day. The TMR was designed to fulfill or exceed the relevant specifications. The dry matter (DM) content was 48.00 %, acid detergent fiber (% of DM) was 18.00%, neutral detergent fiber (% of DM) was 27.00%, non-fiber carbohydrates (% of DM) were 38.00%, crude protein (% of DM) was 17.00%, and net energy for lactation was 1.7% (Mcal/kg). On a five-point scale, cows had an average body condition score of 3.65 ± 0.56.

### 2.3. Experiment Design

Randomly among all cows with SCM, we selected 10 cows with subclinical mastitis and 10 clinically healthy cows (without clinical signs of any diseases) out of 650 milking cows.

Cases belonging to the subclinical mastitis group (SCM) (*n* = 13) were determined based on SCC. Cows with an SCC of more than 200,000 cells/mL were determined to have SCM [23]. SCC was evaluated one time per day during all experiments. According to a general clinical assessment, none of the cows displayed clinical signs consistent with any disease or other factors such as heat stress or estrus. Cows with these factors were excluded from the study (*n* = 3). In this group, the total number of cows was 10.

For the subclinical mastitis treatment protocol, we used an SCC bolus (Groothandel Carton B.V. Julekesweg 7, 7451 PB Holten, Wholesaler in Holten, Netherlands) and nonsteroidal anti-inflammatory drugs (RIMADYL Cattle 50 mg/mL solution) for injection (Zoetis Belgium S.A. Cherrywood Business Park, Loughlinstown Co., Dublin, Ireland). The protocol involved a 90 g SCC bolus, including garlic extract, microcrystalline cellulose, lactose, ethylcellulose, maltodextrin, and magnesium stearate. Cows were treated with one orally administered bolus once. For anti-inflammatory drugs, cows were treated with a single subcutaneous injection at a dosage of 2.5 mL/100 kg body weight.

For the clinically healthy group (HG) (*n* = 10), according Nielen et al. [23], cows were defined as healthy with an SCC of <200,000 cells/mL (average, 158 (±25 cells/mL)). Additionally, these cows did not exhibit any clinical signs consistent with any disease, according to a general clinical assessment.

### 2.4. Measurements

**Figure 1:**
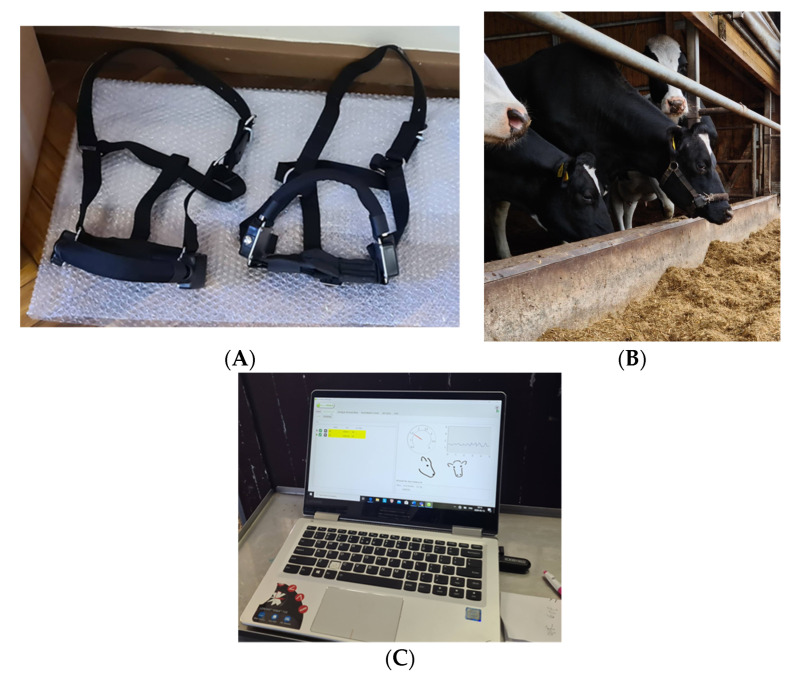
Measurement and data analyses: (**A**) RumiWatch sensors (RWS); (**B**) cows with RWS; (**C**) data visualization and analysis.

Rumination behavior biomarkers were registered with the RumiWatch noseband sensor (RWS; ITIN + HOCH GmbH, Fütterungstechnik, Liestal, Switzerland). Sensors were implanted on the first day after calving. Zehner et al. [20] and Alsaaod et al. [24] developed and confirmed these sensors (Figure 1).

Measurements were from the first day after calving until 13 days after SCM diagnosis. Table 1 summarizes the registered biomarkers.

The RWS consists of a liquid-filled pressure tube and a noseband halter with an integrated pressure detector. In this system, the data logger, which is located on the same halter and enclosed in a safe plastic box, receives a pressure signal from the pressure sensor. Additionally, a sturdy memory cardholder and an acceleration sensor for sensing triaxial head movements are included. At a frequency of 10 Hz, the acceleration values and pressure readings are stored as binary files. The RumiWatch Manager software and the halter are linked by a wireless data transmitter, enabling real-time data collection. Basic algorithms employed within the RWC software process the customized classification of behavioral 10 Hz pressure data characteristics in various time summaries that can be selected. The algorithms classify objects based on the recognition of clear pressure peak clusters produced through jaw motions that are classified according to their behavioral characteristics [20].

### 2.5. Periods of Measurements

In this study, data from the RWS over a period of 13 days before diagnosis and 13 days after the detection of SCM were contrasted against HG data from the same time frame. SCM cows were observed on the same days as the healthy cows.

### 2.6. Data Analysis and Statistics

Hypothesis testing was performed using descriptive statistics to summarize the RumiWatch noseband data (mean and standard deviation). In addition to descriptive statistics, in the hypothesis testing, we used inferential statistics to test the hypothesis of possible differences between groups of HG and SCM cows according to measures of ruminant behavior (rumination time, eating time, drinking time, rumination chews, eating chews, drinking gulps, and boluses).

All statistical analyses were performed using the SPSS 25.0 software (IBM Corp. Released 2017. IBM SPSS Statistics for Windows, Version 25.0. Armonk, NY, USA.). The normal distribution of indicators was confirmed using a Shapiro–Wilk normality test.

The study lasted for a total of 27 days. Days from “−13” to “0” denote experimental period 1 (before the diagnosis of subclinical mastitis at day “0”), whereas days from “1” to “13” indicate experimental period 2 (after the start of treatment).

The study design used repeated measurements, including measurements (time periods) of the same RumiWatch indicator according to the days of the experiment. The mean and standard error of the mean were calculated for the estimated variables. The LSD criterion was used to compare the differences in the mean between group values.

To determine the relationship between subclinical mastitis and rumination behavior registered with an online sensor system, we performed a correlation and logistic regression analysis. The Pearson correlation coefficient was calculated by evaluating the relationships of the studied parameters by groups.

To analyze the factors, multivariable logistic regression models were used. In this process, a backward stepwise logistic model was applied to exclude all non-essential explanatory variables (according to the significance of the Wald test). The estimates and 95% Wald limits were used to calculate the odds ratio (OR) as the ratio of the probability of success to the probability of failure and the 95% confidence interval (CI). The entire final statistical model included only three significant explanatory variables.

All RumiWatch variables (explanatory variables) were grouped into two classes based on the arithmetic mean: class G0 ≤ the arithmetic mean and G1 > the arithmetic mean of all tested cows in the herd four days before mastitis detection in the SCM group of cows (when a significant change in the observed parameters was identified).

For all tests, a probability of less than 0.05 was considered significant (*p* < 0.05).

## 3. Results

The highest probability of mastitis in cows was found at 187.65 ± 2.6 days for milk. On a five-point scale, cows had an average body condition score of 3.65 ± 0.56.

We found that HG cows spent more time engaging in rumination and drinking (*p* < 0.05) and had a higher number of boluses per rumination compared to SCM cows (Table 2). In the period from “−13” to “0” days, rumination time among healthy cows was 10.62% longer (*p* < 0.05); these cows needed 11.90% more rumination chews (*p* < 0.05), their drinking time was 4.83% longer (*p* < 0.05), they had 1.91% fewer gulps, and the number of their boluses per rumination was 2.12% higher compared to SCM cows. Similar trends in the differences in indicators persisted in the second period of the study (from days 1 to 13), but the differences between groups of cows were not statistically significant (Table 2).

On the day of diagnosis, the rumination time of the SCM cows was 39.79% longer, and the drinking time was 16.95% longer; additionally, the number of boluses per rumination was 7.35% higher, but the eating time was 21.84% shorter. Further, the number of eating chews was lower by 14.00%, and that of drinking gulps was lower by 4.34% compared to that of healthy cows (*p* < 0.05).

The activity, downtime, and temperature did not differ significantly between the groups of cows during both periods of the experiment. The uptime of cows in the first period in the SCM group was 5.71% longer, and in the second period of the experiment, uptime was 6.55% shorter compared to that of healthy cows (*p* < 0.05).

**Figure 2:**
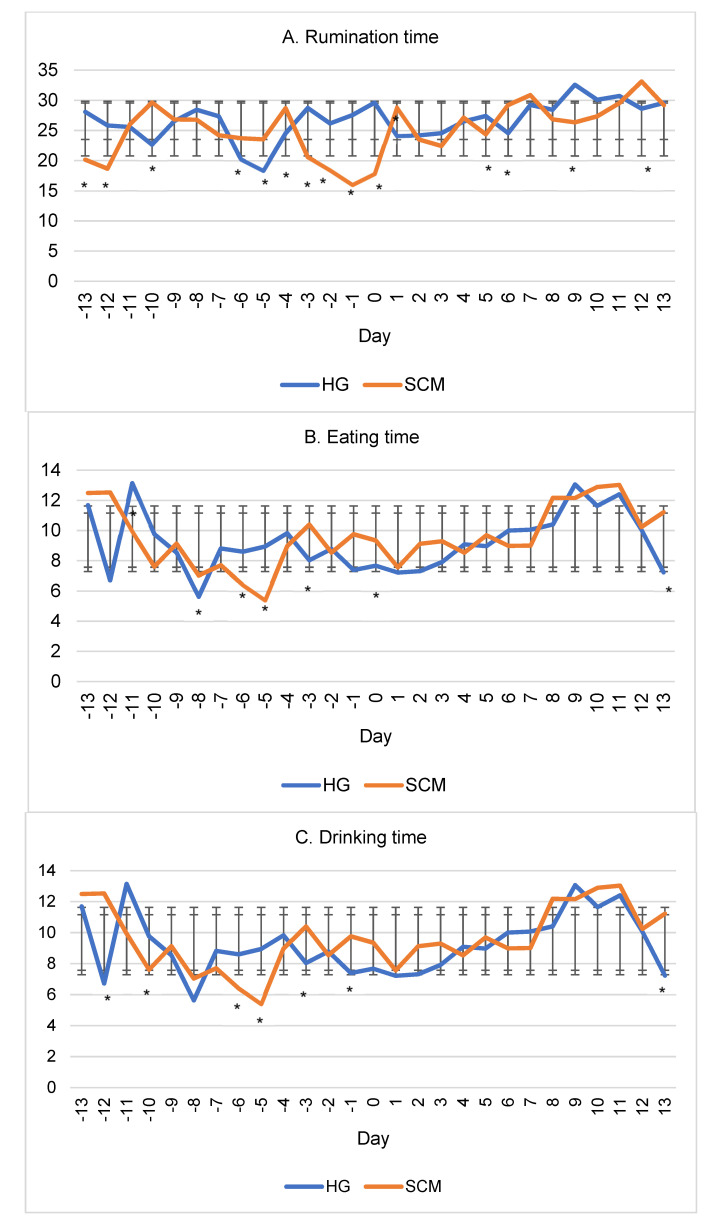

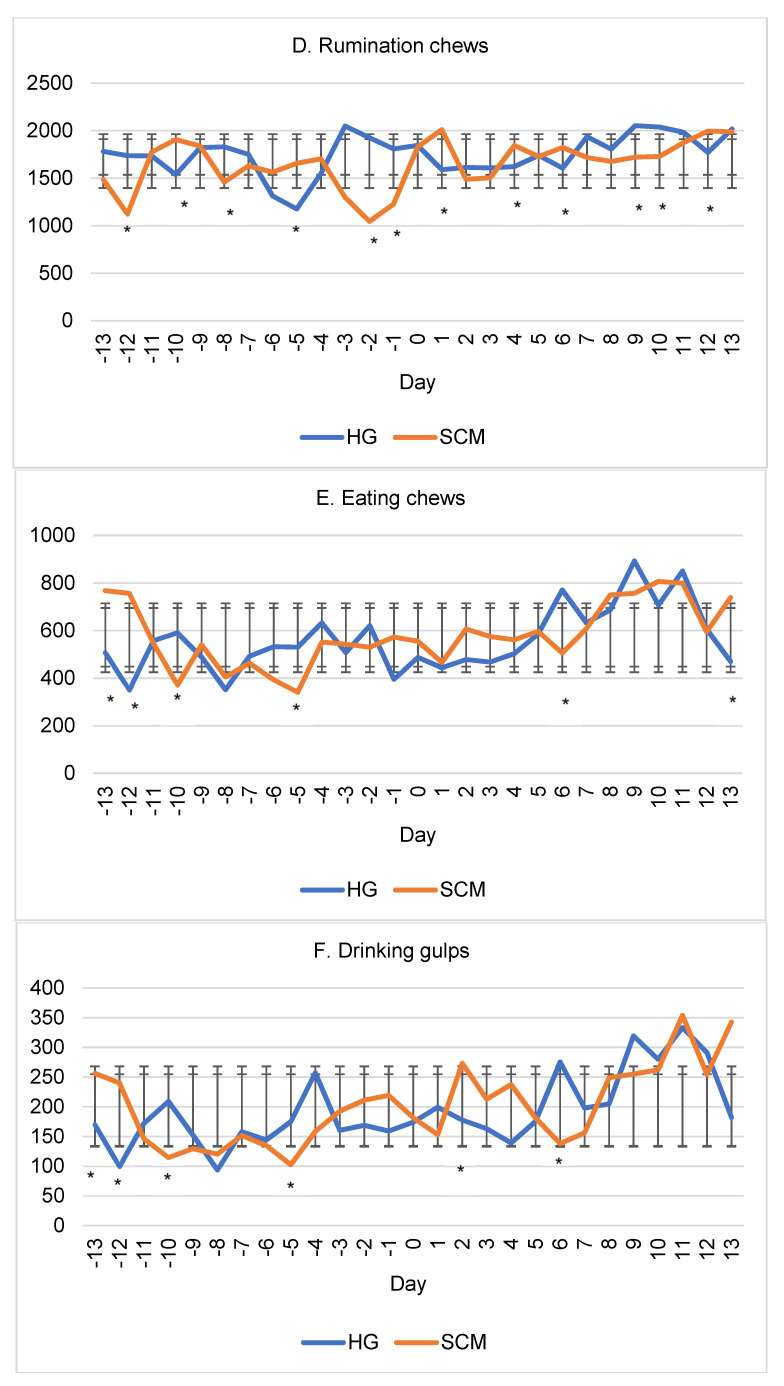

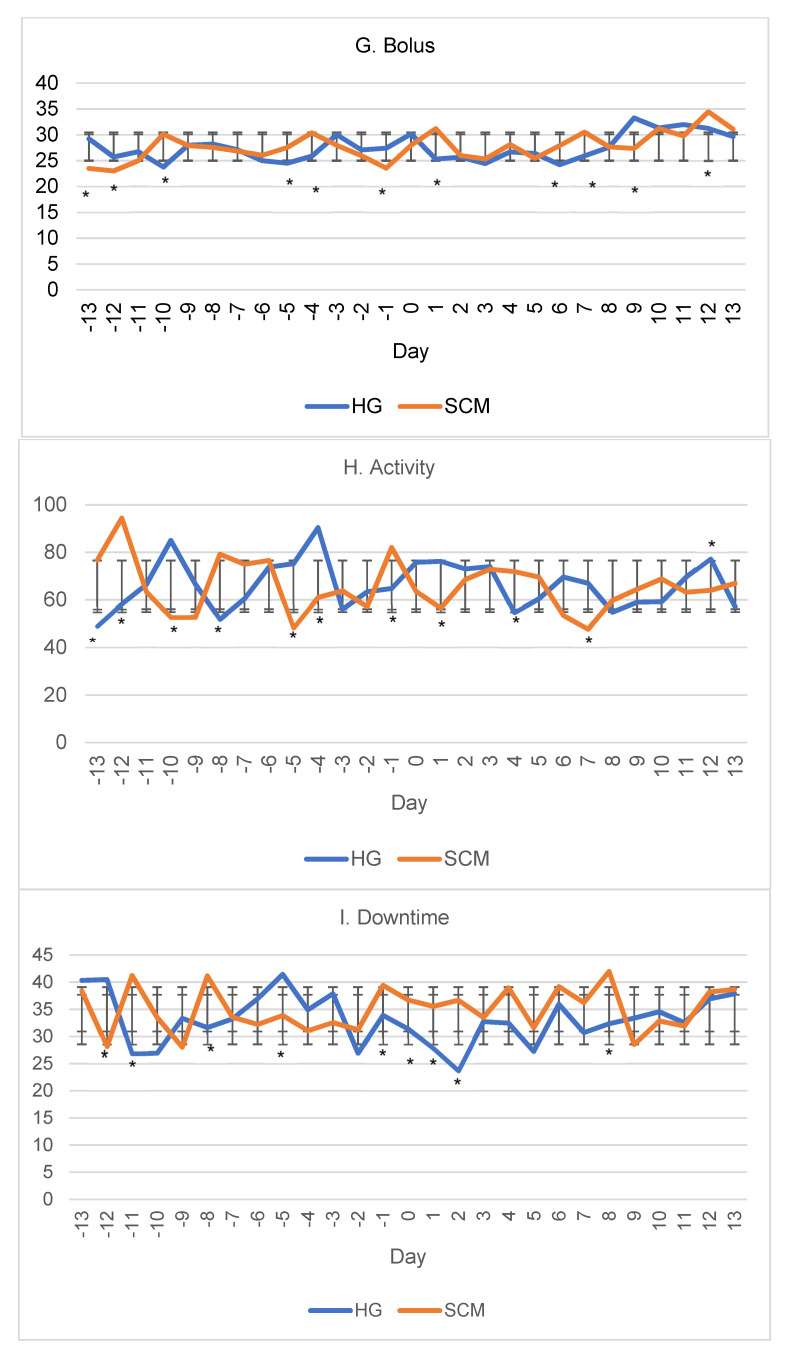

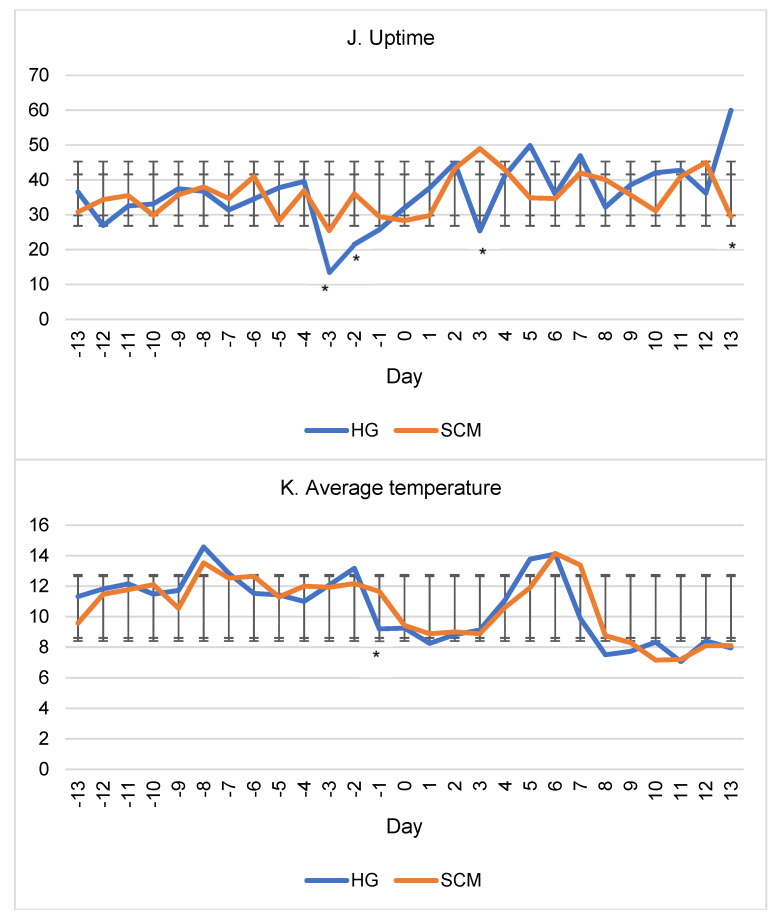
Indicators of cow groups by days of experiment. (**A**). Rumination time; (**B**). Eating time; (**C**). Drinking time; (**D**). Rumination chews; (**E**). Eating chews; (**F**). Drinking gulps; (**G**). Bolus; (**H**). Activity; (**I**). Downtime; (**J**). Uptime; (**K**). Average temperature; HG: clinically healthy group; SCM: subclinical mastitis group (SCM). Error bars indicate standard deviation. The difference between the mean values of the groups is statistically significant at * = *p* < 0.05. The correlation coefficients of the HG group are presented above the diagonal. The group of SCM cows is shown below the diagonal.

Rumination time (Figure 2A) in the SCM group from day −4 to day 0 decreased by 60.91%, drinking time by 48.47 (Figure 2C), and the number of boluses per rumination by 8.67% (*p* < 0.05 (Figure 2G)). On the other hand, during this five-day period, the eating time (Figure 2B) of SCM cows increased by 4.00%, the number of rumination chews (Figure 2D) increased by 6.83%, and drinking gulps (Figure 2F) increased by 12.66% (*p* < 0.05). In the second period experiment, a few significant differences were observed between the SCM group and these indicators of healthy cows (Figure 2A,F).

The average value for the activity of cows in both groups during the second period was slightly lower than that in the first period, and the change in the indicator was unstable throughout the entire period of the experiment (Table 2, Figure 2H). The lowest activity of cows in the SCM group during the first period of the experiment was observed on the fifth day before the onset of the disease and increased up to day −1 (Figure 2H).

The downtime value of SCM cows on the day of disease detection was higher than that of healthy cows (16.22%, *p* < 0.05), and the value of uptime was almost the same for both groups. The change in both of these indicators was unstable and did not show exceptional differences on or before day zero of the experiment (Figure 2I,J).

The average temperature indicator of both groups had a similar trend of change throughout the entire period of the experiment (Figure 2K).

Analyzing the change in the studied parameters during the entire period of the experiment showed uneven changes in cows of both groups. However, in the experimental group, more pronounced changes in most indicators were observed starting from about the fourth day before the diagnosis of the disease.

The rumination time of the cows in both groups was strongly positively correlated with rumination chews and boluses while eating time with correlated with chewing (*p* < 0.01; Table 3).

Rumination chews showed a weak positive association with eating chews and drinking gulps in the HG group (*p* < 0.01), while no significant linear relationship was found between these variables in the SCM group.

A moderate positive correlation was detected between drinking gulps and boluses in the HG group, along with a very weak correlation coefficient in the SCM group (*p* < 0.01).

The drinking time in both groups of cows had a very weak association with all other studied indicators.

Cow activity showed a moderate negative correlation with rumination time in healthy cows and, conversely, a negative correlation in the SCM group (*p* < 0.01).

Downtime showed a weak positive association with drinking time in the HG group and an association in the opposite direction in the SCS group (*p* < 0.05).

Uptime was slightly positively associated with rumination time, drinking gulps (*p* < 0.01), and activity (*p* < 0.05) for HG cows and SCS groups.

Average temperature showed an opposite relationship between all indicators studied in the HG group. However, in the SCM group, we found a weak positive correlation with drinking time (*p* < 0.01).

The odds ratios for the effects of the risk factors on mastitis are reported in Table 4. Mastitis was associated with rumination time, rumination chews, and boluses (*p* < 0.05).

The chosen method of exploring associations using a multivariable table, where one of the dimensions was an outcome of interest with two categories (subclinical mastitis detected or not detected), showed that subclinical mastitis among cows in the herd was more likely with a rumination time of ≤23.80 (OR = 18.271 times, *p* = 0.007) and number of boluses per rumination of ≤27.63 (OR = 15.976, *p* = 0.013) and less likely when the level of chews during rumination (i.e., utilizing molars for the mechanical breakdown of regurgitated items into finer bits) was ≤1627.88 (OR = 0.266, *p* = 0.030) (Table 4).

## 4. Discussion

There is increasing interest in using behavior to detect disease early and, ideally, automatically [20,25]. In this study, we hypothesized that subclinical mastitis affects rumination behavior in cows (such as rumination time, eating time, drinking time, rumination chews, eating chews, drinking gulps, and boluses) registered with sensor systems. The aim of the current study was to determine the relationship between subclinical mastitis and rumination behavior such as rumination time, eating time, drinking time, rumination chews, eating chews, drinking gulps, and boluses, which were registered using online sensors. Based on the results of our study, we found that healthy cows spent more time engaging in rumination and drinking (*p* < 0.05) and had more boluses per rumination. Additionally, it was ascertained that rumination time among cows with subclinical mastitis decreased by 60.91% from day 4 to day 0, while drinking time decreased by 48.47%, and the number of boluses per rumination decreased by 8.67% (*p* < 0.05). Kaufman et al. [26] found that healthy cows spent an average of 19.12 min/h ruminating. In contrast, dairy cows spend approximately 7 h each day engaging in rumination (15.5 min/h) [27]. However, the majority of dairy cows that receive mixed diets ruminate for a shorter period of time [28]. Several studies found a link between reduced rumination time and clinical and subclinical health problems [27,29]. It was discovered that during the period of illness, RT decreased by 5.33% for subclinical mastitis (SCM) and by 14.85% for clinical mastitis (CM), which could be attributed to the difficulties faced by the animals with mastitis [30]. In one study that compared a group of healthy cows to a group of sick cows, there was a significant variation in RT among cows. The RT began to diverge from the normal pattern 2 weeks before the diagnosis of mastitis [16]. Other studies, however, have reported lower RTs in sick animals when comparing cows with health problems to healthy cows [31,32]. When other investigators evaluated behavioral data prior to the manifestation of health problems, the authors discovered that the RT parameter aided in the early detection of diseases before the animals were clinically impacted [32]. Compared to healthy cows, cows with CM were found to have a 397 min/d lower RT [13]. As a result, the large release of those inflammatory mediators that regulate the release of inflammatory cells on the damaged tissue regulated by the hypothalamus, presumably activating the satiety center, could be a feasible explanation for the animals’ reduced average RT [33]. A lower RT is a useful indicator for health risk information and monitoring the metabolic states linked with illnesses [34]. The average RT of multiparous dairy cows was found to predict SM and CM up to 2 days before illness onset. Furthermore, RT variability suggested that animals with subclinical instances fluctuated less when ill [24]. On the day of diagnosis, we found that the rumination time of SCM cows was 39.79% higher than that of the other cows. Additionally, the drinking time was 16.95% longer, and the number of boluses per rumination was 7.35% higher. However, the eating time was 21.84% shorter, and the number of eating chews and drinking gulps was 14.00% and 4.34% less, respectively, compared to the results for the group of healthy cows (*p* < 0.05).

Feeding behavior changes have long been used to diagnose the onset of disease [27]. Although the absence of external signs such as heat, swelling, discoloration of the udder, abnormal secretions, and systemic reactions such as fever and loss of appetite distinguish subclinical mastitis, several studies have revealed behavioral changes several days before clinical signs of illness [35]. Changes in rumination behaviors and ruminating duration on a daily basis, for example, were linked to metabolic problems and discovered several hours or days before clinical signs appeared [17].

Cows with endotoxin-induced acute mastitis demonstrated normal illness behavior, such as decreased appetite. When the most severe clinical signs were present, the cows spent more time eating and less time engaging in rumination and drinking. Furthermore, in contrast to previously documented sickness behavior, cows increased their number of steps [23]. According to various studies, the values of the RumiWatch noseband sensor and locomotion should be analyzed together. The poor appetite observed in sick cows was argued to represent a strategy for decreasing the absorption of some micronutrients required for pathogen proliferation [23]. In the present study, cows ate for extended periods of time, most likely due to fever and illness, but we did not observe the normal sickness behaviors associated with reduced appetite [23]. According to González et al. [36], the eating length among some cows decreases with the development of mastitis, while other cows show no change. Feeding activity in barn-housed cattle is well coordinated, with major peaks in both feeding and social competition coinciding with the fresh food delivery and smaller peaks following milking [37]. Among cattle, water and feed intake are favorably connected [38]. However, drinking has a lower impact on health than feeding, and water is more immediately necessary for body function. For example, adequate feed and water consumption is required to sustain good rumen function [39]. Furthermore, because drinking requires less time than feeding, drinking is less likely to be disrupted by social competition [40]. Additionally, the availability of a sufficient number and length of troughs is important since dominant animals could hinder others from obtaining sufficient amounts of water. Although water consumption was found to be reduced in cows with mastitis [41], King et al. [12] also found reduced walking activity prior to the clinical diagnosis of mastitis. The quadratic associations described here between SCC, and both behavioral changes and distance moved are important since activity may increase in cows with clinical mastitis [42], likely due to udder discomfort and a reduction in laying time. For example, udder pain can hinder animals from lying down and standing up, such that they avoid resting instead of intentionally being more active. According to Jadhav et al. [43], the threshold SCC value to differentiate subclinical mastitis from normal mastitis should be 310,000 cells/mL, rather than 200,000 cells/mL, as currently used [44]. Additionally, the threshold of 200,000 cells/mL for the detection of subclinical mastitis was used in other studies [45,46,47].

We found that the differences in the second period were less significant between the groups, possibly because environmental factors affected the results (e.g., heat stress, estrus, and other effects). Reith et al. [17] revealed that, for estrus identification, the daily rumination time was reduced by 19.6% (83 min/d) on the day of estrus, which could be another explanation for our diverse findings.

Changes in the amount of time a cow spends feeding and ruminating, for example, can reflect an underlying shift in cow contentment and welfare. Furthermore, it is commonly believed that variations in eating and rumination times can assist farmers in forecasting estrus [48]. Davison et al. [24] discovered that cattle displayed indicators of heat stress for extended periods of time during the day when ambient climatic conditions were favorable (in excess of 6 h). This study supports the possibility of incorporating heat stress monitoring into neck-mounted collars by automatically reporting times. According to Weary et al. [49], changes in animal welfare, such as heat stress, can be recognized by a decrease in rumination time. Heat stress has an effect on RT in temperate regions, such as Germany in Central Europe. Moreover, cows modify their RT in response to heat stress thresholds, which are lower in temperate zones than in hotter locations [50]. However, Abeni and Galli [51] reported that the greatest reduction in daylight RT (from 0800 to 2000 h) varied from 49% for cows in the early stages of lactation. In our past study, we found that on the day of lameness identification, reductions in rumination time, eating time, rumination chews, drinking time, and bolus count in the lame group could act as lameness markers [52].

Based on the analysis of all studied risk factors for mastitis, we found that the final statistical model included only three significant explanatory variables: rumination time, rumination chews, and boluses. The results of the model were influenced by the fact that strongly correlated variables were not used in the multiple logistic regression model to ensure the absence of multicollinearity. Moreover, explanatory variables were continuously removed from the model according to the significance of the Wald criterion. Ultimately, cow mastitis is undoubtedly a result of the influence and interactions of many complex factors.

One limitation of our research is that the groups were relatively small. We investigated a subclinical mastitis group (*n* = 10) and a clinically healthy group (*n* = 10). Subsequent studies should increase the number of cows. Additionally, factors such as heat stress, estrus, and other effects that have an impact on rumination behavior biomarkers should be evaluated in a larger study.

As a result, to the best of our knowledge, this is the first study that assesses the relationship between subclinical mastitis and rumination behaviors such as rumination time, eating time, drinking time, rumination chews, eating chews, drinking gulps, and boluses, registered using online sensors.

## 5. Conclusions

Ultimately, subclinical mastitis was found to affect rumination time, and rumination chews registered with the sensor systems. However, additional studies with a larger number of animals are required to confirm these results. Furthermore, the impact of heat stress, estrus, and other effects on rumination behavior biomarkers should be evaluated.

From a practical point of view, changes in rumination behavior could be used to identify subclinical mastitis in dairy cows. Farmers could benefit greatly from this method to recognize affected cows and treat udder inflammation at an early stage. Veterinarians and farmers should consider online sensor systems that register rumination behavior biomarkers for the identification of subclinical mastitis. These systems would allow the early identification of sick cows and reduce the economic losses to farms due to this disease.

Future studies should focus more on factors such as heat stress, estrus, and other effects that can also have impacts on rumination behavior biomarkers; these studies should also use more animals. Additionally, in subsequent studies, the values of RWS and locomotion should be analyzed together.

## Figures and Tables

**Table 1:** Biomarkers of rumination behavior identified using real-time sensors and quantified using the RumiWatch noseband sensor (ITIN + HOCH GmbH, Fütterungstechnik, Liestal, Switzerland) and their descriptions [20].

Parameter	Description
Rumination time (RT)	Time spent on ruminating chews, including chewing breaks of up to 5 s
Eating time (ET)	Time spent chewing food, including breaks of up to 5 s
Drinking time (DT)	Time spent drinking, including delays between gulps of up to 5 s
Rumination chews (RC)	Molars chewing during rumination for mechanical reduction of regurgitated materials into smaller bits
Eating chews (EC)	Total number of trepidation bites and mastication chews made when eating
Drinking gulps (DG)	Total amount of gulps taken while drinking
Bolus (B)	Total amount of gulps taken while drinking

s—seconds.

**Table 2:** Rumination behavior biomarkers registered with real-time sensors measured using the RumiWatch noseband sensor by groups of cows and experimental periods.

RumiWatch NoseBand Indicator	HG	SCM
	Period 1 (−13–0 days)	
	Mean ± standard deviation	Mean ± standard deviation
Rumination time (min/h)	25.70 ±2.02 ^a^	22.97 ± 1.99 ^b^
Eating time (min/h)	8.87 ± 1.94 ^a^	8.91 ± 2.07 ^a^
Drinking time (min/h)	1.24 ± 0.20 ^a^	1.18 ± 0.18 ^b^
Rumination chews (n/h)	1704.54 ± 235.67 ^a^	1501.58 ± 276.92 ^b^
Eating chews (n/h)	504.89 ± 88.02 ^a^	522.19 ± 127.073 ^a^
Drinking gulps (n/h)	164.87 ± 40.03 ^a^	168.03 ± 48.927 ^a^
Bolus (n/rumination)	26.46 ± 1.97 ^a^	25.90 ± 2.30 ^a^
Activity	66.91 ± 2.03 ^a^	67.58 ± 2.77 ^a^
Downtime	34.01 ± 1.05 ^a^	34.36 ± 2.54 ^a^
Uptime	31.37 ± 1.26 ^a^	33.16 ± 2.01 ^b^
Average temperature	11.68 ± 0.99 ^a^	11.62 ± 1.01 ^a^
	Period 2 (1–13 days)	
	Mean ± standard deviation	Mean ± standard deviation
Rumination time (min/h)	27.74 ± 2.03 ^a^	27.59 ± 0.79 ^a^
Eating time (min/h)	9.62 ± 1.91 ^a^	10.33 ± 1.79 ^a^
Drinking time (min/h)	1.17 ± 0.23 ^a^	1.07 ± 0.06 ^b^
Rumination chews (n/h)	1798.68 ± 184.91 ^a^	1776.95 ± 168.97 ^a^
Eating chews (n/h)	621.20 ± 151.34 ^a^	643.63 ± 113.18 ^a^
Drinking gulps (n/h)	226.05 ± 64.61 ^a^	237.37 ± 67.44 ^a^
Bolus (n/rumination)	27.97 ± 3.114 ^a^	28.94 ± 2.70 ^a^
Activity	65.52 ± 2.32 ^a^	63.66 ± 2.44 ^a^
Downtime	32.17 ± 1.01 ^a^	35.68 ± 1.45 ^b^
Uptime	41.06 ± 2.58 ^a^	38.37 ± 1.61 ^b^
Average temperature	9.38 ± 0.72 ^a^	9.57 ± 0.56 ^a^

HG: clinically healthy group; SCM: subclinical mastitis group. ^a,b^: The difference between the mean values of the groups HG and SCM marked with different letters is statistically significant at *p* < 0.05.

**Table 3:** Pearson’s correlation between biomarkers registered with real-time sensors using the RumiWatch noseband sensor by group.

Indicators	Rumination Time	Eating Time	Drinking Time	Rumination Chews	Eating Chews	Drinking Gulps	Bolus	Activity	DownTime	UpTime	Average Temperature
Rumination time	**×**	0.276 **	−0.100 **	0.933 **	0.318 **	0.340 **	0.935 **	−0.460 **	−0.008	0.118 **	−0.353 **
Eating time	−0.021	**×**	−0.176 **	0.205 **	0.798 **	0.687 **	0.328 **	−0.011	0.009	0.114 **	−0.189 **
Drinking time	−0.066 *	−0.037	**×**	−0.087 *	−0.099 **	−0.151 **	−0.164 **	−0.105 **	0.244 **	−0.128 **	0.187 **
Rumination chews	0.779 **	−0.037	−0.119 **	**×**	0.259 **	0.249 **	0.881 **	−0.533 **	−0.066 *	0.018	−0.249 **
Eating chews	0.007	0.960 **	−0.075 *	−0.016	**×**	0.875 **	0.330 **	0.114 **	−0.077	0.201 **	−0.288 **
Drinking gulps	0.010	0.777 **	−0.076 *	0.009	0.844 **	**×**	0.428 **	0.314 **	−0.003	0.271 **	−0.530 **
Bolus	0.766 **	0.025	−0.150 **	0.699 **	0.031	0.133 **	**×**	−0.406 **	0.088 **	0.004	−0.407 **
Activity	0.498 **	0.290 **	−0.088 **	0.490 **	0.270 **	0.306 **	−0.580 **	**×**	−0.203 **	0.009	−0.191 **
Downtime	0.149 **	−0.088 **	−0.201 **	0.224 **	0.068 *	0.047	0.215 **	−0.006	**×**	−0.100 **	−0.020
Uptime	0.321 **	−0.012	0.045	0.072 *	0.092	0.167 **	0.081 *	0.078 *	0.036	**×**	−0.235 **
Average temperature	−0.139 **	−0.569 **	0.127 **	−0.342 **	−0.658 **	−0.747 **	−0.329 **	0.003	−0.077 *	0.065*	×

Statistically significant coefficient of correlation: * = *p* < 0.05; ** = *p* < 0.01.

**Table 4:** Analysis of mastitis risk indicators using a multivariable logistic regression model.

Risk Indicators	Classes of Explanatory Variables	B	S.E.	Wald χ2	df	*p*	OR (95% CI OR)
Rumination time	G0 ≤ 23.80	2.905	1.082	7.203	1	0.007	18.271 (8.756–31.117)
	G1 > 23.80						
Rumination chews	G0 ≤ 1627.88	−1.323	0.609	4.714	1	0.030	0.266 (0.081–0.879)
	G1 > 1627.88						
Boluses	G0 ≤ 27.63	2.771	1.111	6.225	1	0.013	15.976 (7.248–30.223)
	G1 > 27.63						
Constant		−2.283	1.113	4.208	1	0.040	0.102

The classes of explanatory variables were formed according to the average values of herd indicators from day −4 to day 0. B: unstandardized regression weight; S.E.: standard error; Wald χ2: the test statistic for the individual predictor variable; df: degrees of freedom, *p*: *p*-value (statistically significant with a *p*-value < 0.05); OR: odds ratio, 95% CI OR: 95% confidence interval for odds ratios.

## Data Availability

The data presented in this study are available within the article.
